# Supplementary material for: EnzML: multi-label prediction of enzyme classes using InterPro signatures
Source: BMC Bioinformatics. 2012 Apr 25;13:61. doi: 10.1186/1471-2105-13-61 (PMC3483700; doi:10.1186/1471-2105-13-61)
Supplement: Addtional file 5 — The Java code to format the data files, evaluate and predict. The file enzml_java_code.tar.gz contains the Java code used to format database data to ARFF and XML formats, to execute cross and train-test (jackknife) evaluations and to record evaluation results to database. More information is included in the readme.txt file and the Javadoc files. The code can be used with a MySQL database. To use a different database software, other JDBC drivers might be required. [file 1471-2105-13-61-S5.gz › java_code/utils/doc/uk/ac/ed/inf/utils/stats/TruncatedPareto.html]

TruncatedPareto


---


|  |  |  |
| --- | --- | --- |
| **Overview** \| **Package** \| **Class** \| **Use** \| **Tree** \| **Deprecated** \| **Index** \| **Help** | |  |
| **PREV CLASS**   **NEXT CLASS** | **FRAMES**    **NO FRAMES**     **All Classes** |  |
| SUMMARY: NESTED \| FIELD \| CONSTR \| METHOD | DETAIL: FIELD \| CONSTR \| METHOD |  |


---


## uk.ac.ed.inf.utils.stats Class TruncatedPareto

```
java.lang.Object
  cern.colt.PersistentObject
      cern.jet.random.AbstractDistribution
          cern.jet.random.AbstractContinousDistribution
              cern.jet.random.Pareto
                  uk.ac.ed.inf.utils.stats.TruncatedPareto
```

**All Implemented Interfaces:**: DoubleFunction, IntFunction, java.io.Serializable, java.lang.Cloneable

**Direct Known Subclasses:**: PseudoTruncatedPareto

---

``` public class TruncatedPareto extends Pareto ```

A utility class to extract from a truncated Pareto (power-law, long-tail)
distribution. Numbers are extracted from a standard Pareto distribution. If
the number extracted in above the truncation threshold, another extraction is
performed. http://en.wikipedia.org/wiki/Pareto\_distribution

**Version:**
:   19 Jul 2008

**Author:**
:   Luna De Ferrari luna.deferrari-at-ed.ac.uk

**See Also:**: Serialized Form

---

**Constructor Summary**

`TruncatedPareto(double scale,
double shape,
int offset,
int truncationThreshold)`


| **Method Summary** | |
| --- | --- |
| `int` | `getOffset()` |
| `Pareto` | `getPareto()` |
| `java.util.TreeMap<java.lang.Integer,java.lang.Integer>` | `getParetoFrequencies(int numberOfExtractions)`             Extracts numbers from a pareto distribution for the given number of times and records the freqencies with which the integers have been extracted |
| `int` | `getTruncationThreshold()` |
| `int` | `nextInt()`             Returns a random number from the distribution; returns (int) Math.round(nextDouble()). |

**Methods inherited from class cern.jet.random.Pareto**

`cdf, nextDouble, nextDouble, pdf, setState, staticNextDouble, toString`

**Methods inherited from class cern.jet.random.AbstractDistribution**

`apply, apply, clone, makeDefaultGenerator`

**Methods inherited from class java.lang.Object**

`equals, getClass, hashCode, notify, notifyAll, wait, wait, wait`

**Constructor Detail**

### TruncatedPareto

```
public TruncatedPareto(double scale,
                       double shape,
                       int offset,
                       int truncationThreshold)
```

**Parameters:**: `scale` - the distribution scale: `shape` - the distribution shape: `offset` - an offset (all extracted numbers will start from the offset): `truncationThreshold` - a truncation threshold (no extracted number will be above the threshold)


**Method Detail**

### getOffset

```
public int getOffset()
```

---


### getPareto

```
public Pareto getPareto()
```

---


### getParetoFrequencies

```
public java.util.TreeMap<java.lang.Integer,java.lang.Integer> getParetoFrequencies(int numberOfExtractions)
```

:   Extracts numbers from a pareto distribution for the given number of times
    and records the freqencies with which the integers have been extracted

    :   **Parameters:**: `numberOfExtractions` - the number of integer extractions from a a pareto distribution **Returns:**: a map containing an integer as key and a frequency as value (frequency = number of times the integer has been extracted)

---


### getTruncationThreshold

```
public int getTruncationThreshold()
```

---


### nextInt

```
public int nextInt()
```

:   **Description copied from class: `AbstractDistribution`**
:   Returns a random number from the distribution; returns
    (int) Math.round(nextDouble()). Override this method if
    necessary.

    :   **Overrides:**: `nextInt` in class `AbstractDistribution`


---


|  |  |  |
| --- | --- | --- |
| **Overview** \| **Package** \| **Class** \| **Use** \| **Tree** \| **Deprecated** \| **Index** \| **Help** | |  |
| **PREV CLASS**   **NEXT CLASS** | **FRAMES**    **NO FRAMES**     **All Classes** |  |
| SUMMARY: NESTED \| FIELD \| CONSTR \| METHOD | DETAIL: FIELD \| CONSTR \| METHOD |  |


---
